# Myelinating Schwann Cell Polarity and Mechanically-Driven Myelin Sheath Elongation

**DOI:** 10.3389/fncel.2017.00414

**Published:** 2018-01-05

**Authors:** Nicolas Tricaud

**Affiliations:** Institut National de la Santé et de la Recherche Médicale, Institut des Neurosciences de Montpellier, Université de Montpellier, Montpellier, France

**Keywords:** myelin, Schwann cell, peripheral nerves, cell polarity, mechanosensitivity, body growth, YAP, HIPPO pathway

## Abstract

Myelin sheath geometry, encompassing myelin sheath thickness relative to internodal length, is critical to optimize nerve conduction velocity and these parameters are carefully adjusted by the myelinating cells in mammals. In the central nervous system these adjustments could regulate neuronal activities while in the peripheral nervous system they lead to the optimization and the reliability of the nerve conduction velocity. However, the physiological and cellular mechanisms that underlie myelin sheath geometry regulation are not yet fully elucidated. In peripheral nerves the myelinating Schwann cell uses several molecular mechanisms to reach and maintain the correct myelin sheath geometry, such that myelin sheath thickness and internodal length are regulated independently. One of these mechanisms is the epithelial-like cell polarization process that occurs during the early phases of the myelin biogenesis. Epithelial cell polarization factors are known to control cell size and morphology in invertebrates and mammals making these processes critical in the organogenesis. Correlative data indicate that internodal length is regulated by postnatal body growth that elongates peripheral nerves in mammals. In addition, the mechanical stretching of peripheral nerves in adult animals shows that myelin sheath length can be increased by mechanical cues. Recent results describe the important role of YAP/TAZ co-transcription factors during Schwann cell myelination and their functions have linked to the mechanotransduction through the HIPPO pathway and the epithelial polarity factor Crb3. In this review the molecular mechanisms that govern mechanically-driven myelin sheath elongation and how a Schwann cell can modulate internodal myelin sheath length, independent of internodal thickness, will be discussed regarding these recent data. In addition, the potential relevance of these mechanosensitive mechanisms in peripheral pathologies will be highlighted.

## Introduction

Peripheral myelination is a complex process that results in the wrapping and electrical insulation of large axons by myelinating Schwann cells (mSCs) in the peripheral nervous system. Successive myelin segments allow for the efficient and energy cost-effective propagation of axon potentials via the salutatory conduction (Hodgkin and Huxley, 1952). However this requires the optimization of geometrical parameters of the myelin sheath, for instance, regulation of the internodal myelin sheath thickness and length relative to the diameter of the ensheathed axon. Indeed when myelin deposition is too thin or too thick, the generation of axon potentials at the node is less efficient and nerve conduction can fall from its maximum (Rushton, 1951; Waxman, 1997). In addition short internodal distances or, in other words, too short myelin segment lengths relative to the axon diameter, sharply reduces nerve conduction velocity (Waxman, 1980) while increasing this length leads to a flat maximum (Wu et al., 2012). Experimental measures of the myelin sheath thickness and length in different nerves of different species have shown that evolutionary pressure lead to the optimal myelin sheath geometry for a given axon diameter (Rushton, 1951).

The data also demonstrates the strong correlation that exists between myelin sheath thickness and axonal diameter which underlies one of most famous characteristics of peripheral nerves: that on the same axon successive myelin segments all have a very similar length and thickness. The discovery of the role of axonal Neuregulin 1 type III in the determination of myelin sheath thickness in mSC (Michailov et al., 2004; Taveggia et al., 2005) has provided the first mechanistic ground for this axon diameter/myelin sheath thickness correlation. However, axonal Neuregulin 1 type III do not regulate myelin sheath length. Moreover, the correlation between internodal length and the axonal diameter is less clear, indicating other parameters may be involved in internodal length determination.

In term of cell biology these correlations and the geometrical homogeneity of the myelin sheath along the same axon are a real challenge for the myelinating Schwann cell. How does the cell know the length of the myelin segment she has to produce? And then how cells on the same axon end up with the same myelin sheath internodal length? Recent results suggest that part of the answer lies in the strict control of the myelin sheath geometry through sophisticated molecular mechanisms among which mechanotransduction processes. My goal in this article is to review the existing data on the regulation of the myelin sheath internodal length, to discuss the molecular mechanisms that govern mechanically-driven myelin sheath elongation, to hypothesize how a Schwann cell can modulate internodal myelin sheath length, independent of internodal thickness, and to highlight the potential role of mechanosensitive mechanisms in some peripheral nerve pathologies.

## Peripheral Nerve Stretching During Developmental Body Growth Drives Myelin Sheath Elongation

What are the evidences supporting the role of mechano-transduction processes in the determination of myelin sheath geometry? The process of myelination starts late during mammalian nerve development; postnatally in mice or rats and around birth in humans. This is a lengthy process that requires around a quarter of the lifetime of a mouse (6 months) and one fifth of the human lifetime (20 years) to achieve it. A new born organism can potentially be exposed to numerous external changes and insults over this lengthy period. The relative exposure of the nerves to the external world as they reach the skin and epithelia and the relatively weak blood-nerve barrier makes peripheral myelination susceptible to various alterations. A tight control of the process is therefore a guarantee of safety in the production of correct myelin sheath geometry. Moreover, during peripheral nerve myelination, the intensity of the myelination process is not regular. Indeed most of the myelin turns are generated early after myelination starts (Friede and Samorajski, 1968; Webster, 1971). The internodal length does not also extend uniformly during myelination and most of the myelin sheath length is obtained before 1 month after birth in rat and before 10 years in human (Jacobs and Love, 1985; Jacobs, 1988).

Taken together these data indicate that myelin sheath radial and longitudinal growth does not result from a passive deposition of myelin along axons, but instead is the outcome of intrinsic cellular events that are tightly regulated over time and can integrate extrinsic events occurring during the postnatal development of the body (Jacobs, 1988).

One of the major events occurring during postnatal development in mammals is the body growth. As an example femur and tibia growth in humans and rats is geometric between birth and adolescence (Schlaepfer and Myers, 1973; Jung et al., 2011) and, in an extreme manner, blue whale postnatal body growth leads nerves to elongate from 1 m in calves to more than 30 m in adults (Smith, 2009). As most peripheral axons have already reached their targets at birth this elongation mechanism is only driven by stretching due to the body growth (Suter and Miller, 2011). Defined as “passive stretching,” “towed growth,” or “stretch growth of integrated axon tracts” this process has long been recognized as critical for peripheral nerve biology, but little is known about the molecular mechanisms. Nevertheless, it can be mimicked in neuronal cultures placed on two glass or plastic plates that are pulled apart at controlled speed (Pfister et al., 2004). In these conditions axons can elongate by 10 cm at a sustained rate of 8 mm/d (330 μm/h) while maintaining their functions (Pfister et al., 2006). In cultures this growth involves numerous cytoskeletal processes and regulations (Suter and Miller, 2011) and a computer model suggests the functions of mechanosensitive ion channels in the axolemma (Purohit and Smith, 2016).

As a large number of peripheral axons are myelinated what would be the impact of this passive nerves and axons stretching on myelin sheath internodal length? First, maximal internodal length is correlated with the size of the organism. Large animals that show a higher body growth factor have longer maximal internodal length: on average maximal myelin sheath length is around 2.4 mm in cows (Tuczinski and Friede, 1984); 2 mm in humans (Friede et al., 1981); 1.8 mm in cats (Nilsson and Berthold, 1988); 1.4 mm in rats (Jacobs, 1988); and 1 mm in mice (Fernando et al., 2016). However, the minimal myelin sheath length is similar in all species between 0.15 and 0.3 mm (Vizoso, 1950; Jacobs, 1988). The same range of length is found in myelinated segments of Schwann cell/neuron co-culture models. When internodal lengths were compared in nerves of different organs, they were significantly longer in organs that grow a lot (legs or forearms) versus organs that remains relatively shorter (crane or jaws) (Vizoso and Young, 1948; Thomas and Young, 1949; Vizoso, 1950). Finally, experimental analysis of the internodal length in human ventral spinal roots showed that, for a given caliber, internodes were more than two times longer in the long sacral than in the shorter cervical roots. The root elongation factor matched closely the internode elongation factor, but not the fiber diameter (Friede et al., 1981, 1985). These data show that internodal length is directly related to the length of the root or the organ that houses the fiber, suggesting that body growth that elongates peripheral nerves also increases the myelin sheath length during postnatal development.

Such a mechanism is supported by an elegant experiment of Jacobs and Myers consisting in the reduction of the rat hind limbs growth by irradiation at 15 days postnatal (Jacobs and Myers, 1993). Irradiation kills dividing cells in growing bones inducing up to 50% decrease in the tibia length at adult age. No demyelination was seen and the number of myelinated fibers was only slightly reduced. Average axons diameter was increased but internodal lengths were reduced by about a half. The opposite experiment, the artificial elongation of peripheral nerves, has also been realized but exclusively on adult animals. Using orthopedic devices bones were cut and elongated at controlled speed, which stretched peripheral nerves. Minimal distraction, close to the usual nerve tensile strain due to limb movements, was buffered by the wavy disposition of the myelinated fibers buffers (Yokota et al., 2003; Lillie et al., 2017). Beyond that, nerve stretching lead to the elongation of the myelin sheath by up to two times its original size without any change in the axon diameter (Yokota et al., 2003; Abe et al., 2004; Simpson et al., 2013; Fernando et al., 2016). Excessive distraction speed nevertheless lead to paranodal retraction, axonal thinning, and eventually demyelination (Yokota et al., 2003; Abe et al., 2004). Taking together these data show that body growth promotes the elongation of mSC on the axon, which would explain why this myelin sheath elongation is not passive but controlled during nerve development.

However, as mSC are not anchored in the spinal cord and on peripheral tissues such as neurons, it remains difficult to conclude that they are themselves stretched during the body growth or the artificial nerve extension. Nevertheless, the full nerve, perineurium and endoneurium, is stretched during the body growth or artificial nerve extension. So the extracellular matrix that surrounds myelinated fibers is likely to be stretched too. As mSC are bound both to the basal lamina via integrins and dystroglycans complexes (Court et al., 2004, 2009) and to axons via the axo-glial junctions (Bhat, 2003), it is therefore possible that these cells are also receiving mechanical cues during nerve stretching. Interestingly, when a look is given at the proteins, whose inactivation or silencing lead to reduced internodal length *in vivo* (**Table 1**), it can be noted that they are involved in the basal lamina (Laminin 2), the dystroglycans complex (dystroglycan, utrophin, periaxin, AHNAK), the actin cytoskeleton regulation (N-WASP, ROCK) and the mechanotransduction (YAP, FRMD6, Merlin). So myelin sheath elongation is mechanically-driven and may rely on classical mechanotransduction processes (Dupont, 2016).

**Table 1:** Gene mutations and silencing that lead to changes in the internodal length or in the myelin sheath thickness.

Protein	Effect on internodal length	Effect on myelin sheath thickness	Reference
Pals1	-	0	Ozçelik et al., 2010
Crb3	+	0	Fernando et al., 2016
FRMD6	+	0	Fernando et al., 2016
Merlin/NF2	-	0	Denisenko et al., 2008; Mindos et al., 2017
YAP	-	-	Fernando et al., 2016
Dlg1	0	+	Cotter et al., 2010
PTEN	0	+	Cotter et al., 2010; Goebbels et al., 2012
AKT1	-	-	Cotter et al., 2010
AKT2	-	-	Cotter et al., 2010
P120ctn	0	-	Perrin-Tricaud et al., 2007
Laminin 2	-	0	Patton et al., 2008
Dystroglycan	-	0	Court et al., 2009
Utrophin	-	0	Court et al., 2009
Periaxin	-	0	Court et al., 2004
AHNAK	-	0	von Boxberg et al., 2014
N-WASP	-	0	Novak et al., 2011
ROCK	-	0	Melendez-Vasquez et al., 2004
Pex5	-	0	Kleinecke et al., 2017

*-: reduced; +: increased; 0: no effect.*

But, how does this translate in myelinating Schwann cell biology?

## Epithelial Cell Polarization

The question of the myelin segment thickness determination has been explored at the molecular and cellular levels using genetically modified mice and *in vivo* viral transgenesis over the last 15 years. The radial extension of myelin sheath is mainly determined by the expression at the axonal cell surface of the trophic factor Neuregulin 1 type III (NRG1 type III) (Taveggia et al., 2010). Indeed the larger the axon, the more the Schwann is exposed to NRG1 type III, resulting in a thicker myelin sheath (Michailov et al., 2004; Taveggia et al., 2005). This mechanism appears to act in concert with other signaling pathways such as laminin, integrin, and PKA (Ghidinelli et al., 2017). In addition, the myelination stimulation pathway is retro-controlled via Dlg1 and PTEN that inhibits AKT activation (Cotter et al., 2010; Goebbels et al., 2012). Interestingly Dlg1 is an epithelial polarity factor (Walch, 2013) and recently epithelial-like cell polarization during myelination has been shown to be critical to control myelin sheath formation (Masaki, 2012).

Cell polarization is characterized by the localized distribution of proteins and molecules within the cell (Ahmed and Macara, 2016). In the epithelial cell apico-basal polarity organizes the cell along an axis determined on one side by the apical domain, adjacent to the lumen, and on the other side by the baso-lateral domain adjacent to the basal lamina and on the lateral membrane (Mellman and Nelson, 2008; Ahmed and Macara, 2016). In other cell types lacking distinguishable apical and the basolateral domains, a similar organization involving two poles has been documented (Muthuswamy and Xue, 2012). As the molecular mechanisms of these bipolar polarizations are similar, as a general meaning this bipolar organization has been termed apico-basal in reference to the epithelial cell. Such a cell polarization is essential for the development of organs and different tissues of the organism: it allows cells to spatially integrate multiple signals, to build an efficient tridimensional cell machinery and to initiate proper biological responses such as directed cell migration, asymmetric cell division, cell orientation, cell fate determination, and differentiation (Mellman and Nelson, 2008). In particular highly specialized cells, such as neurons, are heavily dependent on the polarization process, which determines axon specification and dendrite formation (Takano et al., 2015).

There are three core regulatory complexes that control apico-basal polarity in mammalian cells: Par3 (Par3/Par6/aPKC), Crb3 (Crb3/Pals1/Patj), and Dlg1 (Scrib/Dlg1/Lgl) complexes (Assémat et al., 2008). The two first complexes are apical while the Dlg1 complex is baso-lateral. The establishment and the maintenance of cell polarization is a dynamic process. Indeed the Par3 complex is the first one to appear and progressively segregates to the apical vs. baso-lateral boundary (McCaffrey and Macara, 2009). The Dlg1 complex appears shortly afterward and the resulting physical and chemical interactions between the two complexes will drive apical and basolateral domain organization. Par3, recruited to the initial site of cell–cell contact, recruits the Par6/aPKC complex. Par6 then inhibits aPKC basal kinase activity. When Par6 further recruits the small GTPase cdc42 this induces a conformational change that releases Par6 inhibition on aPKC. The kinase then binds to and phosphorylates Par3, which dissociates from Par6/aPKC complex allowing aPKC to phosphorylate Lethal giant larvae (Lgl) protein of the Dlg1 complex resulting in its association with Dlg1 and Scrib. The Dlg1 complex then antagonizes the distribution of the Par3 complex to promote the formation of a Par3-free region which is the basolateral domain. Two poles are therefore generated. The formation of the Crb3 complex is promoted by the Par3 complex. Crb3, a short transmembrane protein, interacts with the N-terminus of Pals1, facilitating its interaction with PatJ. This membrane-associated complex then interacts with Par6 to locally stabilize the Par3 complex. This creates an apical membrane domain which can be then engineered by the cell to produce membrane protrusion such as lamellipodiae or microvilli in epithelial cells. It is clear that the Crb3 and Par3 complexes collectively promote the formation and maintenance of the apical domain. Moreover dynamic molecular interactions and enzymatic activities underline that the formation, and also the maintenance, of the polarity domains are not permanent and static events. The complexes act as competitors to favor one domain or the other. Therefore, the maintenance of polarization is the result of a delicate equilibrium between competing factors (Muthuswamy and Xue, 2012; Cerruti et al., 2013).

One of the functions of epithelial cell polarization is to participate to the cellular morphogenesis and therefore to the organogenesis. Indeed, epithelial polarity factors control the cellular distribution of trafficking vesicles and phosphoinositides that determine subcellular characteristics of the bilipidic membranes (Martin-Belmonte et al., 2007; Bornens, 2008; Roignot et al., 2013; Harris, 2017). At the plasma membrane level the lipids and proteins composition is essential to physically organize many subcellular domains such as cilia and microvilli, but also adherens and tight junctions, integrins-basal lamina complexes, and other complexes interacting with the basal lamina or with neighboring cells (Bornens, 2008; Harris, 2017). In addition, cell polarization controls the cell cytoskeleton (Zhu et al., 2017) and in particular the actin cytoskeleton that is intimately linked to the plasma membrane (Harris, 2017). This occurs notably through small Rho GTPase such as RhoA, Rac1, and Cdc42 (Mack and Georgiou, 2014; Couto et al., 2017) but also FERM proteins such as Ezrin, Radixin, Moesin (Pelaseyed et al., 2017). As an example, Crb3 possess a FERM protein binding domain (Gao et al., 2016). The conjunction of the regulation of the plasma membrane nature, the interactions with neighboring cells and with extracellular matrix, and the dynamic of the cytoskeleton allow cell polarity factors to organize the cell morphogenesis and shape (Zhu et al., 2017). This is obvious regarding the critical roles of epithelial polarity factors during the scission of the dividing cells (Cerruti et al., 2013; Roignot et al., 2013) but even more significant regarding the epithelial sheet morphogenesis during development. Indeed basolateral and apical polarization participate to the epithelial cell shape modifications that lead to the formation of the *Drosophila* ovarian border epithelium (Veeman and McDonald, 2016), the *Ciona* notochord (Veeman and McDonald, 2016) and zebrafish posterior lateral line primordium (Hava et al., 2009), and mouse embryo or neural tube as examples (Cearns et al., 2016; Zhu et al., 2017). In mammals numerous *in vitro* 3D models have shown that basic organoid morphogenesis relies on the tridimentional organization of cells thanks to their changes in shape (Roignot et al., 2013). Basolateral and apical domains extensions are modified according to the final shape that is required and the temporal and spatial controls of these events relies partly on the classical epithelial cell polarity factors (Cearns et al., 2016; Hansen et al., 2017; Sidhaye and Norden, 2017). So these factors can change the cell geometry.

## Epithelial-Like Polarization and Geometry of the Myelin Sheath

**FIGURE 1:**
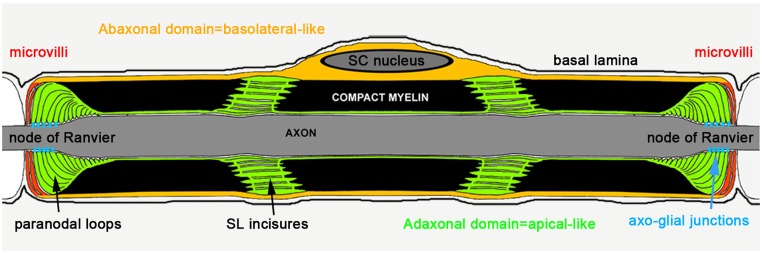
Epithelial-like polarization of the myelinating Schwann cell (Ozçelik et al., 2010).

Epithelial-like cell polarization is essential during Schwann cell myelination and in particular in the control of the geometric parameters evident in the mature myelin sheath (Masaki, 2012). The first evidence of the role of polarity proteins during SC myelination was established in pre/early myelinating cells whereby Pals1, Par3, and LKB1 facilitates the polarization of E-cadherin and p75 receptor in the glial membrane, directly adjacent to the axon (Chan et al., 2006; Shen et al., 2014; Zollinger et al., 2015). This first polarization step is critical to initiate the myelin sheath wrapping of the axon. Epithelial polarity markers, polarity proteins, phospholipids, and trafficking proteins have also been mapped in mature myelinating SC (Ozçelik et al., 2010). Basolateral polarity markers are localized in the abaxonal domain of the mSC (the cytoplasmic domain furthest from the axon and adjacent to the basal lamina, **Figure 1**) while apical polarity markers are found in Schmidt-Lanterman (SL) incisures paranodal loops and adaxonal domain (the cytoplasmic domain adjacent to the axon, **Figure 1**). In addition, Crb3 is expressed in the microvilli that extend from the abaxonal domain of the myelin sheath toward the nodes of Ranvier (**Figure 1**). Taken together, these data indicate that the mSC is polarized in a manner comparable to that seen in epithelial cells. Furthermore, adherens junctions, which are known to delimit the apical and basolateral domains in epithelial cells, are localized in the abaxonal edge of incisures in mSC. As these incisures also contain apical polarity markers, this suggests that incisures and the compact myelin are part of the apical-like domain in mSC. Regarding the role of cell polarity factors in the cell geometry, the epithelial-like polarization of mSC indicates that these factors could be important for myelin sheath geometry.

While these mechanisms have not been extensively studied in mSC, several publications support a central role for cell polarity proteins in Schwann cell myelination. First the silencing of p120ctn, a catenin required to stabilize classical cadherins with cytoplasmic proteins, disturbed the E-cadherin cluster localization in SCs and induced a decrease in the myelin sheath thickness, but not length, *in vivo* (Perrin-Tricaud et al., 2007). As this phenotype is different from the phenotype resulting from adherens junction dysfunction (Tricaud et al., 2005), this suggested the mislocalization of adherens junctions as the cause. To go further, Pals1 was silenced in mSC *in vivo*. This lead to a reduction of the myelin sheath intermodal length, a thinner myelin sheath, a mislocalization of basolateral vesicular markers Sec8 and Syntaxin4 and a dysfunction in the targeting of myelin protein PMP22 in the compact myelin (Ozçelik et al., 2010). This confirmed that apical polarity markers were required for correct myelination and, in particular, for the correct geometry of the myelin sheath. Acute silencing of Dlg1 in mouse mSC induces a radial hypermyelination without any change in the internodal length (Cotter et al., 2010). Dlg1 functions as a negative regulator of myelin sheath thickening by stabilizing PTEN in mSC and PTEN inhibits AKT activation which is activating myelination. Both AKT1 and AKT2 isoforms are required for both the longitudinal and the radial myelin sheath extension (Cotter et al., 2010). Complementary studies have shown that Dlg1 acts as a scaffold that targets Sec8 vesicles via an interaction with Kif13B to abaxonal domains, where myelin is remodeled (Bolis et al., 2009). Dlg1 also interacts with MTMR2, which would act as an inhibitor of myelin deposition (Bolino et al., 2004). Indeed human CMT4B and mouse mutants for MTMR2 show an abnormal myelin deposition (Bolino et al., 2004). In mutant mice defective for MTMR2, the negative regulation of myelin sheath thickness via Dlg1 and PTEN is not functional, resulting in hypermyelination (Cotter et al., 2010). Crb3 silencing in mouse mSC leads to a longitudinal hypermyelination with, myelin sheath internodes as long as 1.5 mm, in comparison to the average maximal internode of around 1 mm in wild-type mice (Fernando et al., 2016). Interestingly, no change is seen in myelin sheath thickness between Crb3 deficient and wild-type internodes.

## Yap Co-Transcription Factor Conveys Peripheral Nerve Stretching Clues into SC Nucleus

Crb3 is active at mSC microvilli and stimulates the HIPPO pathway through FRMD6, which is also located in mSC microvilli (Fernando et al., 2016). Another activation factor of this pathway, Merlin, is ubiquitously expressed in the cytoplasm and its inactivation also leads to shorter internodes (Denisenko et al., 2008; Thaxton et al., 2011). Silencing Crb3 induces myelin sheath over-elongation (Fernando et al., 2016), uncovering that Crb3 normally limits the lateral extension of myelin segments. The HIPPO pathway is a cascade of kinases that leads to the phosphorylation of the co-transcription factors YAP and TAZ (Elbediwy et al., 2016). This pathway and YAP/TAZ are critical in multiple cells types leading to the control of organ size (Yu et al., 2015). As an example, YAP dominant active expression in liver cells of mice leads to a largely oversized liver while YAP deletion reduces biliary duct size and increases hepatocytes sensitivity to stress (Yimlamai et al., 2015). In the mSC, YAP silencing reduced both internodal length and myelin sheath thickness and its presence was necessary to mediate Crb3’s effect on internodal length (Fernando et al., 2016). Complementary studies showed that YAP and TAZ are redundant to control SC proliferation and differentiation, to drive peripheral myelination and to maintain it (Poitelon et al., 2016; Deng et al., 2017; Mindos et al., 2017). However, YAP activity is deleterious for SC remyelination (Mindos et al., 2017). It is striking that YAP/TAZ are involved in so many different events of peripheral nerve biology, from premyelinating SC proliferation in newborn pups to myelin maintenance and regeneration in adult mice. One potential explanation is that YAP/TAZ activity is modulated by different regulators such as Crb3-FRMD6 (Fernando et al., 2016) or Merlin (Mindos et al., 2017) or by different effectors such as Gαs (Deng et al., 2017), integrins (Poitelon et al., 2016), or Egr2 (Fernando et al., 2016; Lopez-Anido et al., 2016) depending on the developmental stage of myelin.

**FIGURE 2:**
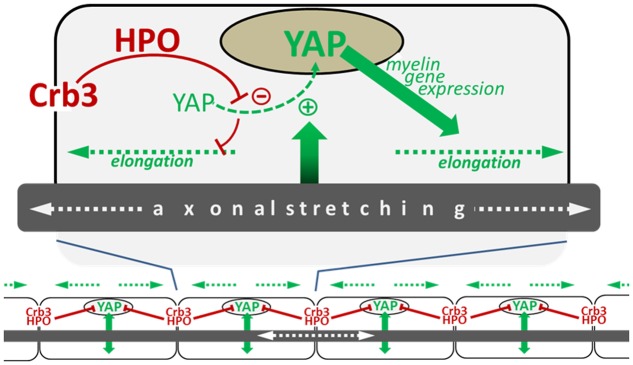
Schematic illustrating the model of myelin sheath elongation in mSC. **(Top)** A myelinating Schwann cell is exposed to input (green arrow) from the stretching axon in the elongating nerve which stimulates the translocation of YAP to the nucleus (green dotted line). YAP nuclear activity promotes myelin gene expression (blue arrow), which increases myelination and myelin sheath elongation (blue dotted arrows). YAP nuclear translocation is negatively regulated by Crb3/Hippo pathway signaling originating from the cells extremities (long red arrow) which phosphorylates YAP, sequestering it in the cytoplasm which consequently limits myelin sheath elongation (short red line). **(Bottom)** The combination of signals promoting YAP activity (green arrows) and inhibiting it (red lines) allows the harmonious growth (blue dotted lines) of all myelin segments along the axon (Fernando et al., 2016).

The amount of active nuclear YAP in mSC is correlated with the efficiency of Crb3 to control internodal length, which is consistent with the role of Crb3 as inhibitor of YAP through the HIPPO pathway. Interestingly, nuclear YAP is also positively correlated with the mouse postnatal body growth; it is highly expressed at early postnatal ages and is decreased later in development, coincident with decreased body growth (Fernando et al., 2016). This suggests that YAP activation, which drives myelin sheath elongation, could directly depend on the intensity of nerve stretching during postnatal body growth. Femoral distraction experiments in adult mice, where the leg nerves are artificially stretched, increased internodal length as seen previously but also increased active nuclear YAP in mSC (Fernando et al., 2016). Silencing YAP in these conditions prevented internode elongation. Furthermore, increasing active nuclear YAP, via the silencing of Crb3, elongated the internodes over what was seen by stretching the nerve alone with intact Crb3. Taking together, these data indicate that nerve stretching during body growth increases internodal length through the activation of YAP in the mSC nucleus, which promotes myelination via transcriptional activity (**Figure 2**).

## An Incremental “Building-Block” Model for Myelin Sheath Longitudinal and Radial Growth

YAP and TAZ are co-transcription factors that interact with transcriptional complexes through TEADs but also other transcription factors such as ErbB4 (Sudol, 2014). In mSC, only the functional interactions with TEADs have been so far demonstrated (Fernando et al., 2016; Lopez-Anido et al., 2016; Poitelon et al., 2016; Deng et al., 2017; Grove et al., 2017). Interestingly, while the transcription of Egr2/Krox20 and most of the major myelin protein genes are modulated by YAP activity (Fernando et al., 2016; Lopez-Anido et al., 2016; Poitelon et al., 2016; Deng et al., 2017; Grove et al., 2017), expression of non-myelin genes such as Rab11a, Integrinα6, Integrinα8, Integrinβ1, Lamininγ1, and α- and β-Dystroglycan are also modified (Fernando et al., 2016; Poitelon et al., 2016; Deng et al., 2017). This suggests that YAP/TAZ activity, and its negative regulation via Crb3-HIPPO pathway, can regulate myelin sheath biogenesis through compact myelin production but also through compact myelin deposition.

This pathway is parallel to the Nrg1-Erb2/3- PI3K-AKT pathway to promote myelination in mSC. Interestingly, this last pathway, together with its negative regulation branch Dlg1-PTEN, has been shown to be critical for the thickening of the myelin sheath (Michailov et al., 2004; Taveggia et al., 2005; Cotter et al., 2010; Goebbels et al., 2012). In contrast, the YAP pathway, together with its own negative regulation branch of Crb3-HIPPO, appears to be more critical for the elongation of the myelin sheath. However, some data suggest that both pathways can interact. Indeed, increasing YAP activity stimulates AKT activity in SC; in turn, increasing AKT activity, in mice that overexpress NRG1 in neurons, increased YAP expression (Fernando et al., 2016). Nevertheless, in these mice the Crb3-HIPPO pathway was sufficient to prevent YAP activation and therefore internodal length did not change significantly (Fernando et al., 2016). This indicates that the Crb3-HIPPO pathway plays a crucial role in dissociating longitudinal myelin sheath extension from radial myelin sheath thickening.

**FIGURE 3:**
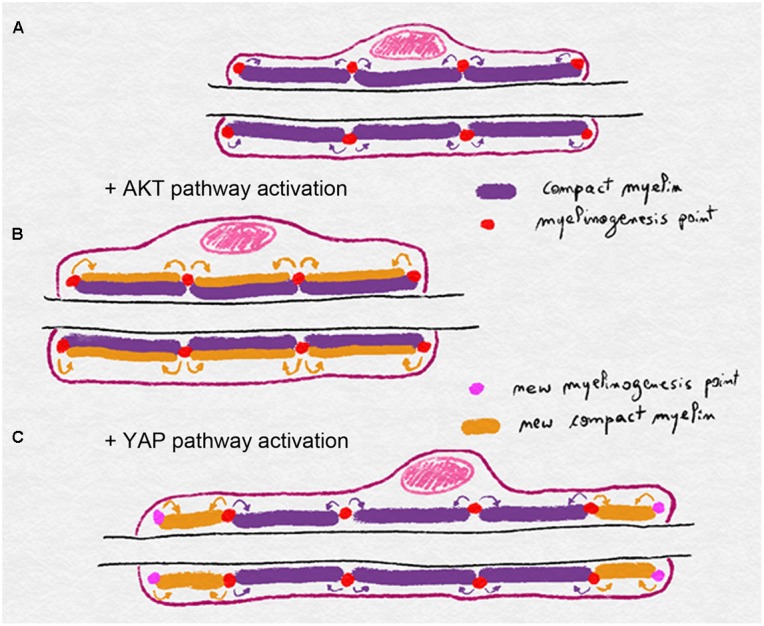
Schematics illustrating the model of myelin biogenesis and deposition in mSC. **(A)** Compact myelin is produced and inserted through myelinogenesis points regularly distributed along the mSC, eventually in incisures and paranodal loops. **(B)** Activation of the AKT pathway stimulates myelin biogenesis and myelin is added radially through the existing myelinogenesis points, leading to a thicker myelin sheath. **(C)** Activation of the YAP pathway stimulates myelin biogenesis but also the formation of new myelinogenesis points along the internode. Compact is then deposited longitudinally leading to a longer myelin sheath.

A model of myelin biogenesis and deposition has been proposed that dissociates longitudinal from radial growth (**Figure 3**). In this model AKT activation, through axonal NRG1 type III, leads to a general myelin biogenesis at a defined number of myelinogenesis points localized at outside edge of the incisures and paranodal loops. These myelinogenesis points are the places where new compact myelin is inserted in the existing one. In this way, production of more myelin, while maintaining same number of myelinogenesis points, results in more myelin turns around the axon without changes in internodal length (**Figure 3**). YAP activation through a hypothetical mechanism that translates the mechanical clues of the nerve stretching also leads to increased myelin biogenesis, but in this case, the addition of new myelinogenesis points along the mSC. As a result, the myelin biogenesis that is coupled to more myelinogenesis points would theoretically result in additional blocks of compact myelin either between two incisures or between the last incisure and the paranodal loops (**Figure 3**). Therefore the combination of more myelin biogenesis and more myelin blocks could lead to the longitudinal deposition of myelin and a longer internode.

## How Mechanical Clues Resulting from Nerve Stretching are Translated into YAP Activation in mSC?

Admittedly, the molecular mechanisms that govern this signal transduction are unknown. However, clues can be found through the analysis of literature and by compiling gene mutations and silencing that lead to changes in the internodal length or in the myelin sheath thickness (**Table 1**). Genes involved in the internodal length regulation are apical polarity factors (Pals1, Crb3), their targets (FRMD6, Merlin) and key myelination biogenesis factors (YAP, AKT1 and 2). Basolateral polarity factors Dlg1, its target PTEN, and the same key myelination biogenesis factors (YAP, AKT1 and 2) are involved in myelin sheath thickness regulation. Interestingly, basal lamina factors (Laminin 2, Dystroglycan) and the factors that participate to the basal lamina signaling in SC (Utrophin, Periaxin, AHNAK) regulate internodal length but not myelin sheath thickness. In addition, actin cytoskeleton regulators (N-WASP, ROCK) have the same properties. As YAP activity is known to be regulated by the stiffness of the extracellular matrix (Elbediwy et al., 2016) and through the actin cytoskeleton, most notably via AMOT and AMOT-like proteins (Moleirinho et al., 2017), these data suggest that mSC basal lamina may be able to modulate YAP activity. This could occur through the plasma membrane complex Dystroglycan-Utrophin-Periaxin that interacts with both the basal lamina and the actin cytoskeleton.

**FIGURE 4:**
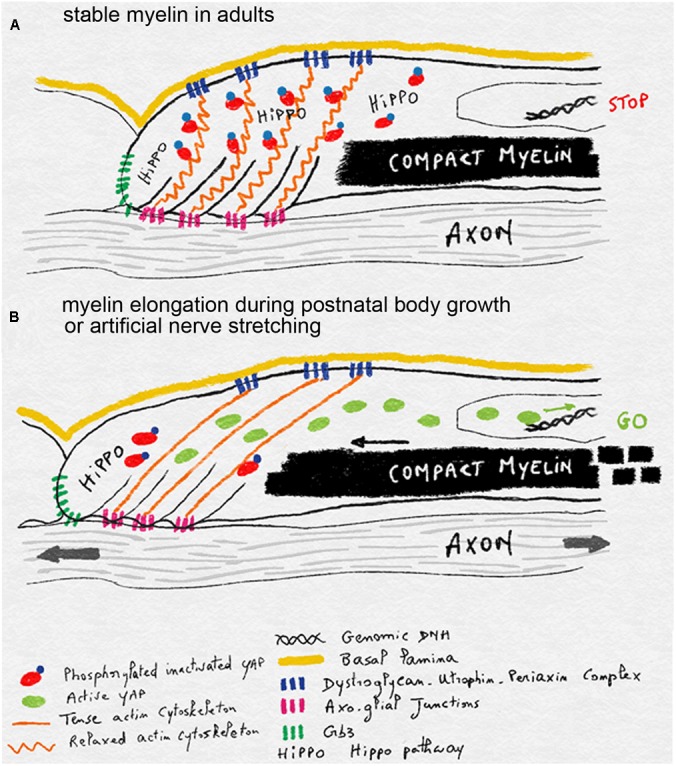
Schematics illustrating the model of mechano-sensitivity in mSC during postnatal body growth. **(A)** In the absence of axonal elongation, in adult nerves, the relaxed actin cytoskeleton anchored to the axon via axo-glial junctions and to the basal lamina via Dystroglycan-Utrophin-Periaxin complexes sequesters YAP. This factor is inactivated through phosphorylation by the HIPPO pathway, which is stimulated by Crb3. Inactive YAP does not reach the nucleus and myelin production and deposition is halted. **(B)** During postnatal body growth or after artificial nerve elongation, axons extend and this stretches the actin cytoskeleton which is also bound to the static basal lamina. YAP is then released from its cytoskeleton prison and can be activated. It enters the nucleus and stimulates myelin production and deposition in order to elongate myelin sheaths.

One hypothesis for the mechano-sensitivity of mSC during postnatal nerve stretching is that axonal extension is detected in the mSC through the tension generated in its actin cytoskeleton. Indeed this cytoskeleton is linked to the axo-glial junctions that attach the mSC to the axon at the level of the paranodal loops (Faivre-Sarrailh and Devaux, 2013). On the outer abaxonal side this cytoskeleton is also linked to the basal lamina through the Laminin-Dystroglycan-Periaxin complex (Sherman et al., 2001) and eventually integrins (Heller et al., 2014). When the axon extends, and if the basal lamina remains static, then actin network could be stretched and in doing so, release the inactivated YAP form. This YAP could then be dephosphorylated and become activated to increase myelin production and longitudinal deposition (**Figure 4**). When body growth and nerve stretching stop then relaxed cytoskeleton could sequester more YAP and the HIPPO activated through Crb3 signaling phosphorylates YAP to slow down myelin production and myelin sheath elongation (**Figure 4**).

## Crossing Node of Ranvier Boundaries

An intriguing question to be raised is whether mSC can grow over each other, crossing the boundaries indicated by the nodes of Ranvier. In the very large majority of nerves, such overlapping never occurs, suggesting a molecular mechanism exists that prevents myelin sheath invasion. Nevertheless this has actually been reported in the sympathetic nervous system of adult mice and rats (superior cervical ganglion in particular) (Kidd and Heath, 1988) and in the spinal roots of new born cats (Berthold and Remahl, 2002). The investigators indicate that this overlapping myelin sheath is quite common at this age and internodal length is very variable. Myelinating cells compete for a piece of axon to myelinate and the losing one can demyelinate and move to another axon and make a second attempt at myelination in a new location (Berthold and Remahl, 2002). This dynamic process ceases a few weeks later as myelin sheath starts to elongate along the axons, resulting in an internodal length that is comparable to other nerves in the adult animal. Taken together this indicates that mSC boundaries exist and are actively maintained during myelination. In some *in vivo* experiments, mSC of the mouse sciatic nerve were infected with viruses expressing Crb3 silencing shRNA together with a fluorescent probe. This leads to sparsely Crb3-silenced fluorescent mSC that over-elongated on their axons, surrounded by non-silenced cells (Fernando et al., 2016). However, these over-elongating myelinating cells never crossed their boundaries with the next cells: nodes were always localized between the myelin segment and overlapping myelin sheaths were never observed, even when examined by electron microscopy. This indicates that despite the loss of Crb3 in microvilli and the disruption of the regulation of longitudinal myelin sheath growth, the boundaries remain intact. Myelinating cells surrounding the over-elongating cell keep their distances, buffer the overgrowth and finally do not change much their size. So Crb3 neither functions as a “cell–cell interaction regulator” and nor do Crb3-silenced cells “win” space over their neighbors. mSC lacking Crb3 just have a higher pace of myelination, independent of the pace of their neighbors. This suggests that homogenous myelin sheath internodal length on the same axon is more likely due to a homogenous longitudinal myelination pace instead of cell–cell competition.

## Pathological Changes Affecting Peripheral Myelin Sheath Internodal Length

One of the most common pathologies affecting peripheral myelination is traumatic nerve injury. Injury induces SC demyelination, which is followed by a remyelination process when the axon has grown back. It has been long known that when SC remyelinate, while they reach their optimal thickness, they never reach their previous internodal length and remain shorter (Jacobs and Cavanagh, 1969; Hildebrand et al., 1986). The reason for this failure is not known but recent data show that, before to remyelinate, repaired Schwann cells are longer than their final size (Gomez-Sanchez et al., 2017). This suggests that extrinsic cues are responsible for the final size of remyelinated cells. The mechano-sensitivity of mSC to peripheral nerve stretching during postnatal body growth can suggest a mechanism: the myelin sheath elongation process requiring YAP and controlled by Crb3-HIPPO is only active when YAP is activated by nerve stretching. In the absence of this stretching, such as during static co-culture myelination, myelin sheath remains appropriately short and close to its starting size. When cells start to remyelinate after a pathologic demyelination occurring in adults there is also no body growth to stretch the nerves and to reengage the earlier YAP driven process, so the myelin sheath internodes do not fully elongate and remain short. In most cases, sections of locally shortened myelin segments are not sufficient to globally impair nerve conduction velocity, so no therapy is needed. However, if remyelination is more generalized, then permanent detrimental outcomes may occur. In Guillain-Barré syndrome, an immune system deregulation induces a massive peripheral nerve demyelination followed by an extensive remyelination. However, despite this remyelination permanent sequels are generally observed (Willison et al., 2016). In this case, a drug or a compound that lift the inhibition of Crb3 on YAP activity may help to restore longer myelin sheath during remyelination.

Some genetic diseases also lead to a reduced internodal length outcome: Periaxin and Laminin 2 (also called Merosin) mutations induce short myelin sheath internodes in peripheral nerves and lead to CMT4F and Lama2 neuromuscular disease respectively (Gillespie et al., 2000; Guilbot et al., 2001; Di Muzio et al., 2003). In both cases the molecular complex that links basal lamina to the mSC cytoskeleton is affected, suggesting this complex is required for internodal elongation during body growth. Recent experiments in mice have confirmed this hypothesis. Mutant Lama2 mouse line mimics the short internodal lengths found in human patients and show an impaired peripheral nerve conduction (Patton et al., 2008). Myelinating cells of these mice have a reduced amount of active YAP in their nuclei with no change in Crb3 expression or in the amount of phosphorylated inactive YAP (Fernando et al., 2016). This suggests that the deficiency in active YAP does not result from more inactivation by the Crb3-HIPPO pathway but more likely from the lack of activation through the mechano-sensitive mechanism during nerve stretching. This is consistent with the model presented previously where the basal lamina, and the complex that links basal lamina to the actin cytoskeleton, is important for the cell to detect the axonal extension during peripheral nerve stretching (**Figure 4**). SCs lacking such a functional complex may be insensitive to the axonal extension and therefore do not extend their myelin sheath correctly. In any case the deficiency in active YAP is indeed one of the causes of the disease; when YAP is activated through the silencing of Crb3 in mutant Lama2 cells the correct internodal is partially restored compared to controls (Fernando et al., 2016).

## Conclusion

Taken together the data presented in this review elaborates on how Schwann cell myelination is controlled, not only through cell polarity factors, but also via cytoskeleton, mechanosensor, junctional proteins, signaling pathways, and co-transcription factors. These pathways act in concert to translate postnatal body growth into an adjusted and optimized myelin sheath internodal length. Of course, many points remain hypothetical and their further study is required in order to provide a clear picture of the myelin sheath formation and the pathological causes of peripheral nerve diseases with short internodes. In addition, as oligodendrocyte myelin sheath internodal length is also critical for the function of the central nervous system (Hamilton et al., 2017), it would be interesting to know whether similar mechanisms are also engaged in this system.

## Author Contributions

The author confirms being the sole contributor of this work and approved it for publication.

## Conflict of Interest Statement

The author declares that the research was conducted in the absence of any commercial or financial relationships that could be construed as a potential conflict of interest.
